# Recent Advance in Ionic‐Liquid‐Based Electrolytes for Rechargeable Metal‐Ion Batteries

**DOI:** 10.1002/advs.202004490

**Published:** 2021-05-02

**Authors:** Wenjun Zhou, Meng Zhang, Xiangyue Kong, Weiwei Huang, Qichun Zhang

**Affiliations:** ^1^ School of Environmental and Chemical Engineering Yanshan University Yanshan Qinhuangdao 066004 China; ^2^ Department of Materials Science and Engineering City University of Hong Kong Hong Kong 999077 China

**Keywords:** aluminum‐ion batteries, ionic liquids, lithium‐ion batteries, magnesium‐ion batteries, potassium‐ion batteries, sodium‐ion batteries, zinc‐ion batteries

## Abstract

From basic research to industry process, battery energy storage systems have played a great role in the informatization, mobility, and intellectualization of modern human society. Some potential systems such as Li, Na, K, Mg, Zn, and Al secondary batteries have attracted much attention to maintain social progress and sustainable development. As one of the components in batteries, electrolytes play an important role in the upgrade and breakthrough of battery technology. Since room‐temperature ionic liquids (ILs) feature high conductivity, nonflammability, nonvolatility, high thermal stability, and wide electrochemical window, they have been widely applied in various battery systems and show great potential in improving battery stability, kinetics performance, energy density, service life, and safety. Thus, it is a right time to summarize these progresses. In this review, the composition and classification of various ILs and their recent applications as electrolytes in diverse metal‐ion batteries (Li, Na, K, Mg, Zn, Al) are outlined to enhance the battery performances.

## Graphical abstract

This manuscript reviews the classification of ionic liquids, and their potential application as electrolytes in metal‐ion batteries (Li, Na, K, Mg, Zn, Al). Their merits of nonflammable property, thermal stability, and high safety suggest that they could be a promising solution to realize high safety and high energy density for next generational battery systems.

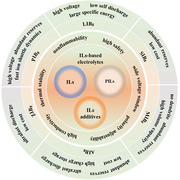

## 1 Introduction

Energy is becoming one of the most challenging issues in the 21st century due to the use‐up of fossil source and the motivation to pursue energy independence and a cleaner environment. Currently, the research of energy mainly has two directions: generation and storage. Alternative energy generations such as solar cells, water splitting, tide, and wind have been widely developed. However, the progress in energy storage seems slightly lagged behind although this field currently is a very hot research topic. Energy storages can be divided into several types including thermal storage, fuel storage, batteries, supercapacitors, etc. Among all storage systems, batteries, as important energy carriers of energy storage, possess the advantages of high efficiency, application flexibility, and fast response speed.^[^ ^1^ ^]^ Now, batteries play indispensable roles in the energy storage market and other practical applications. With the continuous progress of society and the urgent requirement of clean energy and friendly environment, rechargeable metal‐ion (Li, Na, K, Mg, Zn, Al, etc.) batteries with long lifetime, high energy/power density and safety are preferred and attract much attention.^[^ ^2^, ^3^, ^4^, ^5^, ^6^, ^7^, ^8^, ^9^, ^10^, ^11^, ^12^, ^13^, ^14^ ^]^ Although rechargeable lithium‐ion batteries (LIBs) have been demonstrated to show high energy density, the shortage of Li resource has become a potential limitation for future applications. Therefore, other alternative metal‐based rechargeable batteries are being regarded as possible substitutes for LIBs.

As one of crucial parts in rechargeable batteries, electrolytes play an important role in shipping electrons between the cathodes and anodes, which are necessary to endow the batteries with high voltage, high specific energy, long cycling life, high safety, etc. Thus, the strong pursuit of more secure, stable, and high‐performance electrolyte systems is highly desirable. Currently, two electrolyte systems, solid‐state electrolytes and ionic liquids (ILs), are becoming hot research focuses, where solid‐state electrolyte system has several advantages such as low flammability, excellent flexibility, wide electrochemical stability window, superior thermal stability, no leakage, and high safety.^[^ ^15^, ^16^, ^17^, ^18^ ^]^ However, their disadvantages are obvious: poor conductivity, less active electrochemical interfaces, and higher interfacial resistance. The usage of solid polymer electrolytes with special structures might provide a solution to address these issues.^[^ ^19^ ^]^

**Figure 1:**
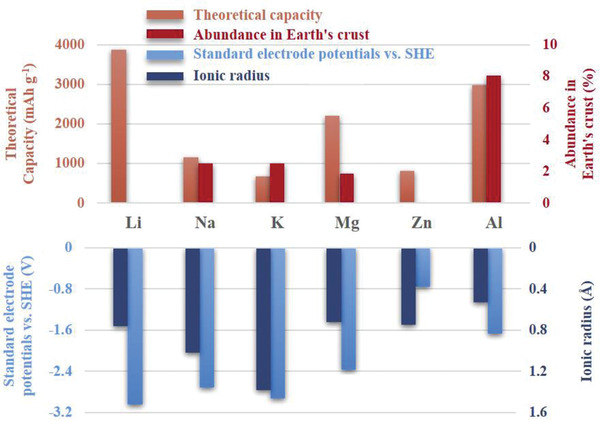
Several natural features of Li, Na, K, Mg, Zn, and Al elements.

ILs, a new type of liquid electrolytes, are organic salts (or inorganic–organic hybrid salts) in liquid forms containing charge‐balanced anions and cations. Their electrochemical characteristics can be modulated through changing the combinations of anions and cations without upsetting the balance.^[^ ^20^, ^21^ ^]^ They are widely considered as one of the most promising green solvents^[^ ^22^, ^23^ ^]^ for various applications such as catalysis,^[^ ^24^, ^25^ ^]^ separation science,^[^ ^26^, ^27^, ^28^ ^]^ synthesis (or reaction medium),^[^ ^29^, ^30^, ^31^, ^32^, ^33^, ^34^ ^]^ electrochemistry,^[^ ^35^, ^36^, ^37^ ^]^ and energy storage.^[^ ^38^, ^39^, ^40^, ^41^, ^42^ ^]^ The wide liquid phase range, high heat resistance, low vapor pressure, wide electrochemical windows, and high ionic conductivity make them highly desirable as electrolytes in various battery systems (i.e., LIBs, sodium‐ion batteries (SIBs), potassium‐ion batteries (PIBs), magnesium‐ion batteries (MIBs), zinc‐ion batteries (ZIBs), aluminum‐ion batteries (AIBs), metal–air,^[^ ^43^, ^44^, ^45^ ^]^ metal–sulfur,^[^ ^46^, ^47^, ^48^, ^49^ ^]^ and metal–oxygen^[^ ^50^ ^]^). Since different metals have different ionic radius, electrode potentials and theoretical capacities, which dramatically affect their performance in batteries with ILs as electrolytes, we have summarized these properties in **Figure** **1** for later clear discussion.^[^ ^51^, ^52^ ^]^

In this review, we mainly summarize the recent progress in the applications of ILs as electrolytes in various rechargeable metal‐ion batteries. After briefly introducing the types of IL‐based electrolytes, the different strategies to improve the diverse battery performance by employing different ILs electrolytes are presented in detail. Moreover, the challenges with respect to ILs as electrolytes in high‐performance batteries are provided in the conclusion.

## 2 Compositions and Types of ILs

**Figure 2:**
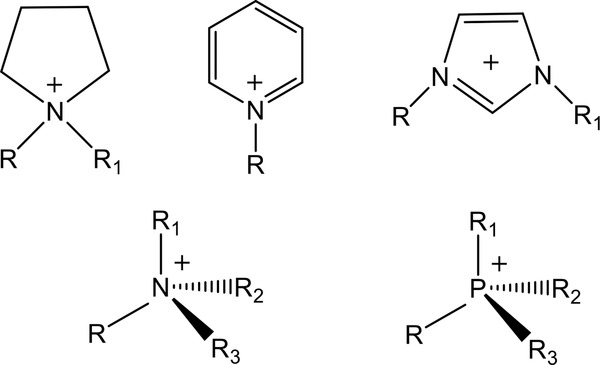
Structure diagrams of [PYrr]^+^, [PYri]^+^, [RRIm]^+^, [NR*_x_*H_4−_ *_x_*]^+^, and [PR*_x_*H_4−_ *_x_*]^+^ cations.

**Figure 3:**
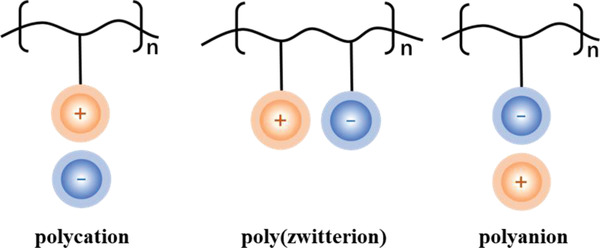
Three types of PILs including PCILs, PZILs, and PAILs.

Since ILs were first synthesized by Walden^[^ ^53^ ^]^ in 1914, many ILs with different combinations of anions and cations have been developed. In this review, ILs will be classified into small molecular ILs and polymer ILs. For small molecular ILs, they generally have large‐size low‐symmetric organic cations and small‐sized inorganic anions. The representative anions are TFSI^–^, FSI^–^, BF_4_^−^, PF_6_^−^, SbF_6_^−^, AsF_6_^−^, C_4_F_9_SO_3_^−^, CF_3_SO_3_^−^, (CF_3_SO_2_)_2_N^−^, CF_3_COO^−^, C_3_F_7_COO^−^, (C_2_F_5_SO_2_)_3_C^−^, (C_2_F_5_SO_2_)_2_N^−^, etc. The cations usually are [PYrr], [PYri], [RRIm], [NR*_x_*H_4−_ *_x_*], or [PR*_x_*H_4−_ *_x_*] types, whose structures are shown in **Figure** **2**.^[^ ^54^ ^]^ Another type of small molecular ILs are AlCl_3_‐type ILs, which were first reported in 1992.^[^ ^55^ ^]^ The two types are all characterized by low vapor pressure, nonvolatility, the adjustability of polarity, wide liquid phase range, high inherent conductivity, wide electrochemical window, as well as dual solvent and catalyst functions. The main difference between the two types of ILs is that the compositions are basically fixed and stable to water and air for the former one, while the latter is extremely sensitive to water and air, and they must be handled in vacuum or inert dried gas atmospheres. Poly(ionic liquids) (PILs) are prepared through the polymerization of IL monomers (anions and cations groups as the repeating units). By introducing diverse anions, cations or both of them on the polymer backbone, the PILs can be divided into three types (polycation ILs (PCILs), polyanion ILs (PAILs) and poly(zwitterion) ILs (PZILs) to realize different functional applications (**Figure** **3**).^[^ ^56^ ^]^ PILs have excellent properties of both ILs and polymers, which could provide several advantages such as flexibility, more safety, and nonleakage.^[^ ^57^, ^58^, ^59^, ^60^ ^]^ The properties of ILs and PILs would ensure the rechargeable batteries to obtain high output voltage, extremely stable cycling performance, high energy and power densities, and to make the batteries more durable and safer.

## 3 Applications of ILs in Various Battery Systems

The conductivity and electrochemical stability of ILs are two important factors to evaluate their performance as electrolytes. ILs exhibit higher viscosity and lower conductivity than organic electrolytes due to the strong Coulombic interactions between cations and anions in the case of controlled variables. To the best of our knowledge, the increase in the volume of ions will increase the viscosity of ILs but lead to the decreased conductivity and vice versa.^[^ ^81^ ^]^ Besides, the conductivity also decreases with the addition of other salts. Interestingly, the use of high‐dielectric‐constant solvents for the dilution of ILs can not only enhance the solubility of salts, but also reduce the association of cations and anions, resulting in the enhanced conductivity of ILs. Temperature also has a great influence on the viscosity and conductivity of ILs. As temperature rises, the viscosity drops and electrical conductivity increases.^[^ ^81^ ^]^ Consequently, it is not surprising to investigate that batteries with ILs electrolytes can show better electrochemical performance at high temperature. The conductivities of some commonly used ILs in batteries are summarized in **Table** **2**.

**Table 1:** Abbreviations and full names of some commom cations and anions

Abbreviation	Full name
[PYrr]^+^	*N,N′*‐Alkylpyrrolidinium
[PYri]^+^	*N*‐Alkylpyridinium
[RRIM]^+^	*N,N′*‐Dialkylimidazolium
[NR*_x_*H_4−_ *_x_*]^+^	Alkylammonium
[PR*_x_*H_4−_ *_x_*]^+^	Alkylphosphonium
[PY13]^+^	1‐Propyl‐3‐methylpyrrolidinium
[PY14]^+^	1‐Butyl‐3‐methylpyrrolidinium
[PY12O1]^+^	1‐Methoxyethyl‐3‐methylpyrrolidinium
[EMIm]^+^	1‐Ethyl‐3‐methylimidazolium
[BMIm]^+^	1‐Butyl‐3‐methylimidazolium
[PMIm]^+^	1‐Propyl‐3‐methylpyrrolidinium
[VEIm]^+^	1‐Vinyl‐3‐ethylimidazolium
FSI^‐^	bis(fluorosulfonyl)imide
TFSI^‐^	bis(trifluoromethylsulfonyl)imide
TfO^‐^	trifluoromethylsulfonate
BF_4_^‐^	tetrafluoroborate

**Table 2:** Viscosity and conductivity of several commonly used ILs at room temperature

ILs	Viscosity [mPa s]	Conductivity [mS cm^−1^]	Ref.
[EMIm]BF_4_	32	16.01	^[^ ^61^ ^]^
[EMIm]TFSI	34	10.8	^[^ ^62^ ^]^
[EMIm]FSI	19	17.74	^[^ ^63^ ^]^
[PMIm]TFSI		1.4	^[^ ^64^ ^]^
[EMIm]TfO	45.7	8.5	^[^ ^65^ ^]^
[PY13]FSI	39	9.14	^[^ ^63^ ^]^
[PY13]TFSI	61	3.9	^[^ ^66^ ^]^
[PY14]TFSI	85	2.2	^[^ ^66^ ^]^
[PY12O1]TFSI	63	5.26	^[^ ^67^ ^]^
[PY12O1]FSI	42.3	7.66	^[^ ^67^ ^]^
N_2224_TFSI	156	1.94	^[^ ^67^ ^]^
0.7 M LiFSI‐[EMIm]FSI	25.5	11.3	^[^ ^63^ ^]^
0.7 M LiFSI[PY13]FSI	52.1	5.8	^[^ ^63^ ^]^
0.3 M LiTFSI‐[PY13]TFSI		1.63	^[^ ^68^ ^]^
0.3 M NaTFSI‐[PY13]TFSI		1.65	^[^ ^66^ ^]^
0.3 M NaTFSI‐[PY14]TFSI		1.8	^[^ ^66^ ^]^
0.2 M LiTFSI‐[PY14]TFSI		1.6	^[^ ^69^ ^]^
1 M NaBF_4_‐[PY14]TFSI		1.9	^[^ ^70^ ^]^
1 M NaN(CN)_2_‐[PY14]TFSI		1.5	^[^ ^70^ ^]^
1 M NaClO_4_‐[PY14]TFSI 1 M NaPF_6_‐[PY14]TFSI		1.0 0.5	^[^ ^70^ ^]^ ^[^ ^70^ ^]^
NaCl‐buffered AlCl_3_/[EMIm]Cl		9.2	^[^ ^70^ ^]^
0.75 M NaBF_4_‐[EMIm]BF_4_		11.8	^[^ ^71^ ^]^
1.1 M NaFSI‐[EMIm]FSI		8.5	^[^ ^71^ ^]^
0.7 M NaTFSI‐[EMIm]TFSI		3.9	^[^ ^71^ ^]^
0.98 M NaFSI‐[PY13]FSI		3.6	^[^ ^71^ ^]^
0.2 M KFSI‐[PY13]FSI	78.2	4.8	^[^ ^72^ ^]^
1 mol kg^−1^ KFSI‐[PY13]FSI	71.7 (30 °C)	3.31 (30 °C)	^[^ ^73^ ^]^
SBA−15/LiTFSI‐[PYRA_12O1_]TFSI/PVDF‐HFP		0.25	^[^ ^74^ ^]^
LiTFSI‐[BMIm]BF_4_/PVDF‐HFP/PC/EC		5.26	^[^ ^75^ ^]^
0.2 M Zn(TFSI)_2_‐[EMIm]TFSI/PVDF‐HCP		1.05	^[^ ^76^ ^]^
SiO_2_PPTFSI/PVDF‐HFP		0.64	^[^ ^77^ ^]^
PVDF‐HFP‐PEO‐ILZE		16.9	^[^ ^78^ ^]^
0.33 M AlCl_3_‐[EMIm]Cl		9.07	^[^ ^79^ ^]^
AlCl_3_–urea‐[EMIm]Cl (13.5:9:0.8)		3.4	^[^ ^80^ ^]^

### 3.1 Small ILs in Different Batteries

#### 3.1.1 Lithium‐Ion Batteries

LIBs are the most widely used battery systems and their success in the field of consumer electronics and electric vehicles has been witnessed.^[^ ^82^, ^83^, ^84^, ^85^, ^86^, ^87^, ^88^, ^89^, ^90^ ^]^ At present, the high energy density of LIBs requires to cramp more Li^+^ into a limited space. However, since Li is an alkali metal and is chemically reactive, such high Li density in a limited space would bring some uncertainties in safety. Thus, how to stabilize the chemical safety of both electrodes is the key issue for LIBs.

**Figure 4:**
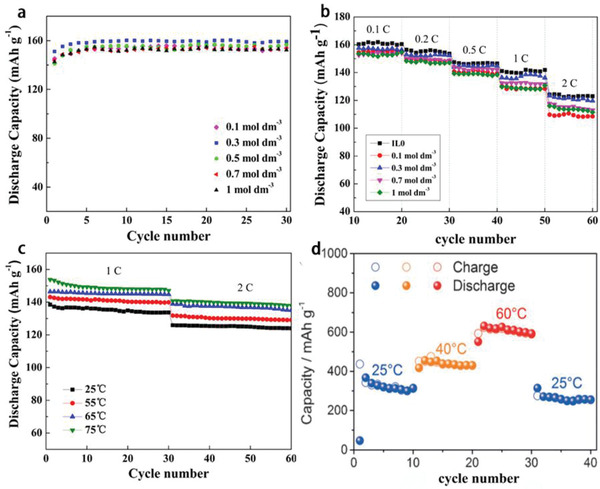
a) Specific discharge capacity, b) cycling behavior of LiFePO_4_‐based LIBs with various LiTFSI concentrations IL electrolytes at room temperature, and c) cycling performance of the LIBs in the IL electrolyte with 0.3 m LiTFSI at different temperatures.a‐c) Reproduced with permission.^[^ ^99^ ^]^ Copyright 2014, Elsevier Ltd. d) Cycling behavior of the Sn–C‐based LIBs with LiTFSI‐[PY14]TFSI electrolyte measured at 25 mA g^−1^ current density at 25, 40, and 60 °C.Reproduced with permission.^[^ ^69^ ^]^ Copyright 2016, Wiley‐VCH.

To realize safe large‐scale energy‐storage LIBs, the usage of ILs as electrolytes in LIBs could be a good choice^[^ ^91^, ^92^, ^93^, ^94^, ^95^, ^96^ ^]^ because ILs as electrolytes have several advantages including easy synthesis, thermostability, relatively high ionic conductivities, and so on.^[^ ^69^, ^97^, ^98^ ^]^ In the previous report, LiTFSI‐[PY13]TFSI with EC/DMC‐5%VC organic additives (ethylene carbonate (EC), dimethyl carbonate (DMC), and vinylene carbonate (VC)) was used as electrolyte for LiFePO_4_‐based inorganic LIBs. The effects of various LiTFSI concentrations on the electrochemical performance of the batteries were studied. Although the number of Li^+^ in the electrolyte increased with the increase of LiTFSI concentration, the viscosity of the electrolyte subsequently also increased. The LIBs showed the best cycling and rate performance at 0.3 m LiTFSI concentration (**Figure** **4**a,b).^[^ ^99^ ^]^ Benefiting from the decreasing viscosity and increasing conductivity of IL electrolytes with the increase of temperature, as well as the extremely low flammability of ILs, the batteries with IL electrolytes have been demonstrated to display excellent performance and promising applications at relatively high temperatures (Figure 4c,d).^[^ ^69^, ^99^ ^]^

**Figure 5:**
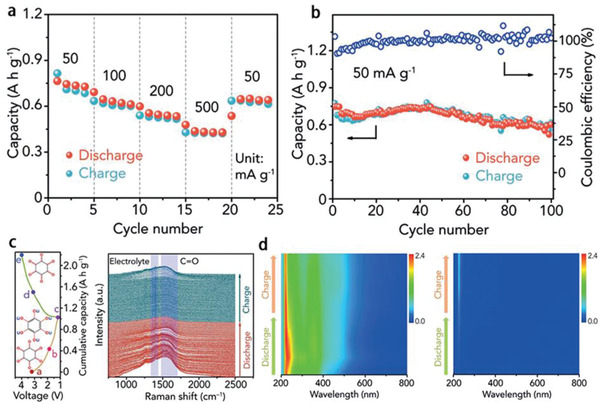
a) Rate performance of C_6_O_6_ at different current densities, b) cycling performance of C_6_O_6_ at 50 mA g^−1^ in 0.3 m LiTFSI‐[PY13]TFSI, c) in situ Raman diagram of C_6_O_6_ charge–discharge process, and d) color‐mapped profiles of ex situ UV–vis spectra in 1 m LiTFSI‐DOL/DME electrolyte and 0.3 m LiTFSI‐[PY13]TFSI electrolyte, respectively. Reproduced with permission.^[^ ^101^ ^]^ Copyright 2019, Wiley‐VCH.

**Figure 6:**
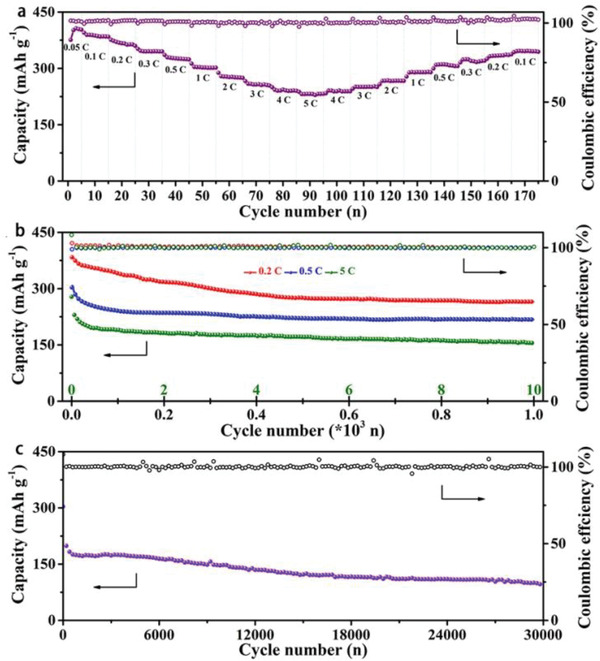
Electrochemical performances of C6Q: a) rate performance at different current densities, cycling stability over b) 1000 cycles (0.2 C, 0.5 C, 5 C) and c) 30 000 cycles (10 C)(1 C = 446 mA g^−1^). Reproduced with permission.^[^ ^68^ ^]^ Copyright 2020, Elsevier B.V.

More importantly, they have been demonstrated to display more obvious effects on electrochemical performances of organic batteries, typically, decreasing the solubility of organic electrodes comparing with that in conventional electrolytes.^[^ ^100^, ^101^, ^102^ ^]^ For example, the Chen group^[^ ^101^ ^]^ reported C_6_O_6_ (cyclohexanehexone) with a high theoretical capacity of 957 mAh g^−1^. With 0.3 m LiTFSI‐[PY13]TFSI and LiTFSI‐DOL/DME (1,3‐dioxolane (DOL) and 1,2‐dimethoxyethane (DME)) as electrolyte, two kinds of LIBs have been fabricated respectively and the electrochemical performances of C_6_O_6_ as electrode have been compared. In the IL electrolyte, C_6_O_6_ not only showed favorable rate performance (**Figure** **5**a), but also displayed high discharge capacity and retention. The capacity retention of C_6_O_6_ in LiTFSI‐[PY13]TFSI electrolyte was about 82% at 50 mA g^−1^ after 100 cycles (Figure 5b), which was much higher than that in traditional LiTFSI‐DOL/DME electrolyte. Figure 5c is the charge–discharge process of C_6_O_6_ in LiTFSI‐[PY13]TFSI, and the vibration strength of electrochemical active C=O bond tended to decrease (lithiation) first and then increase (delithiation). In the color‐mapped profiles of ex situ UV–vis spectra (Figure 5d), the strong adsorption peaks existed in LiTFSI‐DOL/DME electrolyte during the chemical redox process, while no obvious peaks appeared in LiTFSI‐[PY13]TFSI electrolyte, indicating the dissolution‐inhibiting effect of LiTFSI‐[PY13]TFSI toward C_6_O_6_. These results prove the applicability of ILs in promoting the electrochemical performance of LIBs. Similarly, in 2020, the Huang group^[^ ^68^ ^]^ applied the same IL electrolyte into another organic LIBs with Calix[6]quinone (C6Q, *C*_theo_ = 446 mAh g^−1^)^[^ ^103^ ^]^ cathode. The as‐fabricated batteries showed superb rate property and cycling stability over 1000 cycles and even 30 000 cycles at high current density of 10 C (4460 mA g^−1^) (**Figure** **6**). Due to the similar polarities, some small organic molecules are easily soluble in conventional organic electrolytes.^[^ ^104^, ^105^, ^106^ ^]^ These outstanding research results have further verified the vital role of IL‐based electrolytes, especially the inhabitation effects on the dissolution of organic electrodes by polarity differences.

#### 3.1.2 Sodium‐Ion Batteries

Since SIBs are widely considered as one of the greatest potential successors to LIBs for large‐scale applications, they have received substantial research interests recently.^[^ ^107^ ^]^ Na is the next smallest and lightest alkali metal comparing with Li in size and weight. It is also the most common alkali metal element on Earth. Na can supply an electrochemical redox potential of 2.71 V (vs the standard hydrogen electrode (SHE)), which is just 0.3 V less negative than that of Li, resulting in appropriate high operating battery voltage.^[^ ^108^, ^109^, ^110^ ^]^ Nevertheless, because the ionic radius of Na^+^ is the biggest among these metal ions in Figure 1, the embedding/de‐embedding resistance of Na in anodes and cathodes is large. In addition, Na is also very difficult to embed/de‐embed in graphite. Thus, SIBs are suffered with reversibility and irreversible capacity loss. Also, the urgent demand on low cost and high energy density require SIBs to make the batteries safer for various applications.^[^ ^59^, ^111^, ^112^ ^]^ To address these issues in SIBs, ILs as the electrolytes have been widely considered as a promising solution.^[^ ^113^, ^114^, ^115^, ^116^, ^117^, ^118^, ^119^, ^120^, ^121^ ^]^ For example, in 2018, the Chen group^[^ ^66^ ^]^ employed NaTFSI‐[PY13]TFSI as electrolyte in organic SIBs with calix[4]quinone (C4Q) as the electrode, and found that the solubility of C4Q would be strongly suppressed in ILs comparing to normal DME electrolyte and the battery performances were largely enhanced. Besides, some other ILs with FSI^−^/TFSI^−^ anions and [PYrr], [PYri], [RRIm], [NR*_x_*H_4−_ *_x_*] or [PR*_x_*H_4−_ *_x_*] cations have also been employed as electrolytes in SIBs to realize high energy density, where a high redox potential of ≈5 V was achieved.^[^ ^71^ ^]^

In addition to the above‐mentioned ILs, the AlCl_3_‐type IL electrolytes also have excellent electrochemical performance in SIBs. For example, in 2019, the Dai group^[^ ^70^ ^]^ reported a high safety SIB system with AlCl_3_‐type ILs (i.e., NaCl‐buffered AlCl_3_/[EMIm]Cl) as electrolytes with two significant additives: EtAlCl_2_ (ethylaluminum dichloride) and [EMIm]FSI. Such complicated electrolyte has a much higher ionic conductivity (up to 9.2 mS cm^−1^ at room temperature) than some previously reported non‐AlCl_3_‐type ILs.^[^ ^116^, ^122^, ^123^, ^124^ ^]^ The typical action mechanism between anions and anions in AlCl_3_‐based ILs are shown below

(1) $$\mathrm{AlC}l_{3}+\left[\mathrm{EMIm}\right]\mathrm{Cl}\to {\mathrm{AlCl}}_{4}^{-}+{\left[\mathrm{EMIm}\right]}^{+}$$

(2) $$\mathrm{AlC}l_{3}{+\mathrm{AlCl}}_{4}^{-}\to Al_{2}{\mathrm{Cl}}_{7}^{-}$$

**Figure 7:**
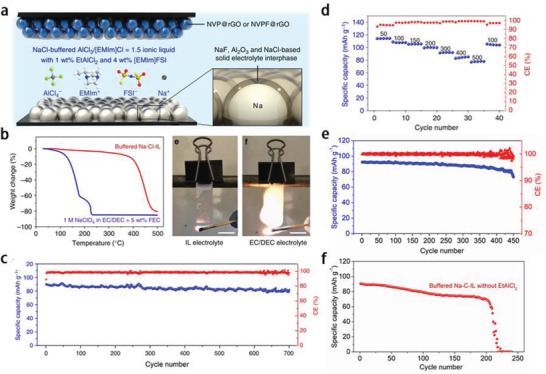
a) Schematic illustration of the battery configuration and electrolyte composition of the IL electrolyte, b) TGA and flammability tests toward Na–Cl–IL and NaClO_4_‐EC/DEC/FEC electrolytes, c) cyclic stability of SIBs with Na–Cl–IL electrolyte at 300 mA g^−1^, d) capacity and Coulombic efficiency of SIBs with Na–Cl–IL electrolyte under current densities from 50 to 500 mA g^−1^, e) cyclic stability of SIBs with NaClO_4_‐EC/DEC/FEC electrolyte at 150 mA g^−1^, and f) cyclic stability of SIBs with Na–Cl–IL electrolyte without EtAlCl_2_ additive at 150 mA g^−1^. Reproduced with permission.^[^ ^70^ ^]^ Copyright 2019, Springer Nature.

In this SIB system, two additives EtAlCl_2_ and [EMIm]FSI (from 1 to 4 wt%) played the critical roles in stabilizing the solid electrolyte interface (SEI) and realizing highly reversible Na plating/stripping on Na anode. The NaF is formed by the reaction of FSI^−^with highly reactive Na, which is the major F‐based SEI component, while Al_2_O_3_ and NaCl are other two leading components of SEI component (**Figure** **7**a). Besides, some other small amount of substances (i.e., Na_2_O, Na_2_SO_4_, and Al) could jointly participate in the composition of SEI. The uniform SEI film can prevent the passage of solvent molecules and avoid the damage caused by the co‐insertion of solvent molecules into electrodes, thus greatly improving the electrochemical performance and battery life.^[^ ^125^ ^]^ The as‐fabricated SIBs with NVP (sodium vanadium phosphate)/NVPF (sodium vanadium phosphate fluoride) as cathodes could obtain a high discharge voltage about 4 V, and high‐capacity efficiency up to 99.9%. The thermogravimetric analysis indicated that the Na–Cl–IL can stable up to 400 °C, while in 1 m NaClO_4_‐EC/DEC/FEC (*V*_EC_:*V*_DEC_ = 1:1, DEC: diethyl carbonate; FEC: fluoroethylene carbonate, 5 wt%), the rapid weight loss began at 132 °C was observed and only ≈15% was retained at 230 °C. Besides this, the Na‐Cl‐IL electrolyte is much safer than the conventional organic electrolyte due to its nonflammable property (Figure 7b). Furthermore, the cycling performance of SIBs with these two electrolytes show big differences. The SIBs with IL electrolytes exhibited good rate performance, reflecting in the excellent Coulombic efficiency from 95% to 99% under 50 to 500 mA g^−1^, high‐capacity retention ratio (more than 90% of the initial specific capacity was maintained after 710 cycles at 300 mA g^−1^), and high‐capacity efficiency (around 98.5%) (Figure 7c,d). By contrast, the capacity retention of SIBs with 1 m NaClO_4_‐EC/DEC/FEC as electrolyte was only 79% after 450 cycles at 150 mA g^−1^ (Figure 7e). Such performance improvement of SIBs with Na‐Cl‐IL as electrolyte is attributed to the cooperation of various components in the IL electrolyte. In the absence of EtAlCl_2_ additive, the capacity dropped rapidly after 200 cycles at 300 mA g^−1^. In the presence of EtAlCl_2_, it could react with H^+^ and NaCl to generate AlCl_4_^−^, C_2_H_6_, and Na^+^ to promote the cycling stability of SIBs (Figure 7f). This type of IL electrolytes with the merits of nonflammability and high conductivity are promising to be applied in SIBs and other rechargeable batteries.

#### 3.1.3 Potassium‐Ion Batteries

**Figure 8:**
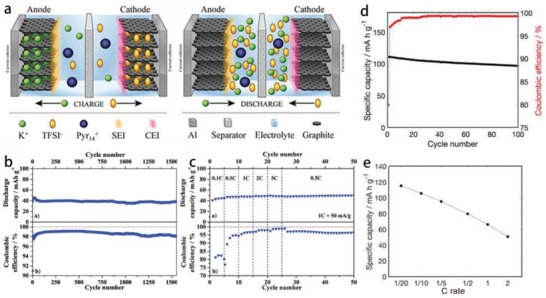
a) Working principle of K‐DGB, b) long‐term cycling performance at 250 mA g^−1^, and c) rate performance at different current densities. a‐c) Reproduced with permission.^[^ ^130^ ^]^ Copyright 2017, The Royal Society of Chemistry. d) Charge–discharge capacity and Coulombic efficiency of KMF/KFSI‐[PY13]FSI/graphite PIBs (cycles 1–5 at C/20, 6–400 at C/5, 1 C = 155 mA g^−1^), and e) rate performance at different current densities. d,e) Reproduced with permission.^[^ ^73^ ^]^ Copyright 2020, American Chemical Society.

PIBs have attracted tremendous attention due to their abundant reserves and low cost.^[^ ^110^ ^]^ The standard electrode potential of K (−2.93 V) is much similar to that of Li (−3.04 V). The lower electrode potential means that it is possible to have a higher energy density, thus makes PIBs more advantageous in high voltage output. In addition, K^+^ can be reversibly embedded in commercial graphite, whereas Na^+^ cannot. PIBs can realize high‐power densities because of the fast migration rate of K^+^.^[^ ^6^, ^126^, ^127^ ^]^ Nevertheless, the severe side reactions between electrolytes and K electrodes may cause an unstable solid‐liquid interface and low Coulombic efficiency. Thus, choosing excellent electrolytes may be the key to the success of PIBs. As new‐type K salts, imides (KFSI, KTFSI) have been proposed to replace traditional ones. The KFSI‐based electrolyte is favorable to form a stable SEI layer, which ensures the PIBs possessing much more stable cycling performance and long lifespan than that with other K salts electrolytes.^[^ ^128^ ^]^ Liu et al.^[^ ^129^ ^]^ proved that the interaction between K^+^ and TFSI^−^ has a good compatibility with the Al current collector at high salt concentration. Yamamoto et al.^[^ ^72^ ^]^ reported [PY13]FSI as electrolyte for high‐voltage PIBs with the voltage of 5.72 V and the ionic conductivity of 4.8 mS cm^−1^ (0.2 m KFSI‐[PY13]FSI) at 25 °C. In 2017, Beltrop et al.^[^ ^130^ ^]^ developed a novel K‐DGB (K^+^‐based dual‐graphite battery) system with 0.3 m KTFSI‐[PY14]TFSI+2 wt% ES (ethylene sulfite) as electrolyte. The working principle of K‐DGB is shown in **Figure** **8**a. During the charge, K^+^ and TFSI^−^ were intercalated into the anode and cathode, respectively, and then came back to electrolyte during the discharge. The battery delivered a discharge capacity of 42 mAh g^−1^ with the Coulombic efficiency near 99% at 250 mA g^−1^ (Figure 8b). Moreover, the capacity is little affected by the increasing current densities, proving its excellent rate property (Figure 8c). Fiore et al.^[^ ^73^ ^]^ combined the potassium manganese hexacyanoferrate cathode and graphite anode with the KFSI‐[PY13]FSI electrolyte. By optimizing the morphology of KMF (K_2_Mn[Fe(CN)_6_)]) and using IL electrolyte, the PIBs obtained a high capacity (119 mAh g^−1^), long cycle life of retaining 87.4% after 100 cycles at 0.1 C (15.5 mA g^−1^) and capacity retention of 43% at 2 C (310 mA g^−1^) (Figure 8d,e). An important but heavily underestimated advantage is that the Coulombic efficiency achieved 99.3% for KMF cathode due to the stability of the KFSI‐[PY13]FSI electrolyte under high potential and the inhibition of corrosion toward Al current collector.^[^ ^73^ ^]^ All above advantages make the developments of PIBs more attractive and practical. The study on cathode materials of PIBs is still in its early stages, and it is urgent to develop suitable materials to enhance energy density of PIBs. Up to now, there are relatively few reports on ILs electrolytes in PIBs, the optimization of electrolytes is the primary task in developing high‐performance PIBs.

#### 3.1.4 Magnesium‐Ion Batteries

As one of the “post‐LIBs”, MIBs have attracted wide attention due to their excellent safety (no dendrite), low price and high volumetric energy capacity (3833 mAh cm^−3^). However, there are still many problems to be overcome before their successful commercial usage. These issues include how to develop new electrolytes that enable Mg anode to charge and discharge steadily, how to design and prepare highly stable electrode materials, and how to solve the low voltage problem. In LIBs, the SEI can protect the anode and reduce side reactions,^[^ ^131^ ^]^ whereas, such knowledges cannot be used in MIBs directly. Recent studies have shown that conventional electrolytes (i.e., Mg(ClO_4_)_2_, Mg(TFSI)_2_, or Mg(PF_6_)_2_ salts) in carbonate (or ether solvents) work poorly in MIBs due to the generation of SEI layer on the surface of Mg anode that can passivate both ions and electrons.^[^ ^132^ ^]^ Although reversible Mg deposition and stripping could be enhanced in Grignard reagents (RMgX, R = alkyl or aryl group, X = halide in ethereal solvents), their intrinsic poor oxidative stability severely limits their further applications.^[^ ^133^ ^]^ Even though, still a large number of electrolytes (i.e., carborane‐based electrolytes) have been demonstrated to show high oxidative stability.^[^ ^134^ ^]^

**Figure 9:**
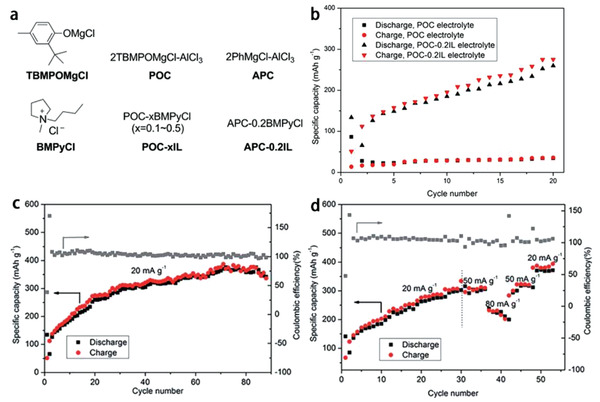
Electrochemical performances of MIBs with CoS_HE_ cathode in 0.25 m POC‐0.2IL electrolyte: a) chemical structures of electrolytes or electrolyte components, b) the cycling process within 20 cycles of CoS_HE_ cathode in POC electrolyte and POC‐0.2IL electrolyte, c) cycling performance at a current density of 20 mA g^−1^, and d) rate performance at the current densities ranging from 20 to 80 mA g^−1^ in POC‐0.2IL electrolyte. Reproduced with permission.^[^ ^135^ ^]^ Copyright 2019, The Royal Society of Chemistry.

To realize reversible high‐capacity MIBs, the Zou group^[^ ^135^ ^]^ proposed two novel strategies: 1) the design of a 3D fractional porous cobalt sulfide (CoS) micron sphere with large pore volume (0.227 cc g^−1^), considerable specific surface area (27 m^2^ g^−1^), high theoretical capacity (589 mAh g^−1^) and high structural flexibility; 2) the usage of [PY14]Cl IL as electrolyte additive to promote the phase transformation of CoS during the charge–discharge processes and change the kinetic properties and thermodynamic of CoS and Mg conversion, for activating the charge–discharge process and enhancing the capacity for reversible MIBs. Besides, some additives in electrolytes can efficiently improve the electrochemical performance of batteries.^[^ ^136^ ^]^ For example, in the 0.25 m POC‐0.2IL (POC: 2TBMPOMgCl‐AlCl_3_; TBMPOMgCl: 2‐(*tert*‐butyl)‐4‐methylphenolate magnesium chloride) electrolyte (**Figure** **9**a), the IL additive of [PY14]Cl is helpful to inhibit Cl^−^ corrosion at high potentials.^[^ ^137^ ^]^ The low Coulombic efficiency in the earlier cycling stage (Figure 9b) may be ascribed to the formation of SEI that hindered the recharge process and led to the capacity loss. Then the electrode was activated gradually. The activation speed of MIBs with the POC‐0.2IL electrolyte was much faster than that with only the POC electrolyte, indicating the obvious lifting effect of the additive [PY14]Cl on the electrode activation process. The MIBs displayed a stable cycling ability of around 340 mAh g^−1^ over 88 cycles at 20 mA g^−1^ and considerable rate property of about 300 mAh g^−1^ at 50 mA g^−1^ (Figure 9c,d).

**Figure 10:**
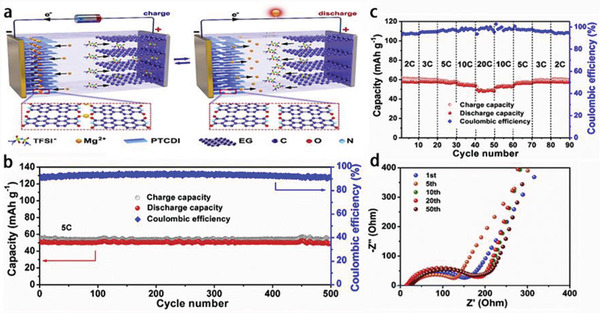
a) Schematic diagram of the working mechanism of Mg‐DIB‐based on the PTCDI/EG, b) long‐term cycling stability at 5 C (500 mA g^−1^) for 500 cycles, c) rate performance and corresponding Coulombic efficiencies at various current densities from 2 C (200 mA g^−1^) to 20 C (2000 mA g^−1^) and d) Nyquist plots at 1st, 5th, 10th, 20th, and 50th cycles of the Mg‐DIBs. Reproduced with permission.^[^ ^138^ ^]^ Copyright 2020, Elsevier B.V.

Unlike the nature of rapid transit for Li^+^ between anodes and cathodes, Mg^2+^ is much slower in shuttle rate due to its higher charges, and the stronger binding force with the cathode materials causes the collapse of cathode material structures, and leads to the poor reversible charge–discharge performance of MIBs. In 2020, Lei et al.^[^ ^138^ ^]^ changed the traditional research by adjusting the anode and cathode materials and developed a new type of Mg‐based dual‐ion battery (Mg‐DIB), where the IL containing Mg salt was used as the electrolyte (0.4 m Mg(TFSI)_2_‐[PY14]TFSI), expanded graphite (EG) was used as anode material. A small organic molecule (3,4,9,10‐perylenetetracarboxylic diimide, PTCDI) was employed as cathode material because its slightly solubility in the IL electrolyte and good Mg storage property. Such arrangement has changed the traditional appearance of electrodes based on inorganic materials. The working mechanism of the Mg‐DIBs is that, during charge, the anions in electrolyte intercalate with the graphite while Mg^2+^ in electrolyte are stored in organic small molecule PTCDI, and during discharge, the anions and cations in electrode materials return to electrolyte (**Figure** **10**a). By combining with the working principle of double ions reaction, the problem of slow and irreversible reaction of Mg^2+^ in cathode materials was well solved and the high output voltage of MIBs (>4.5 V) was improved. This research proved that Mg(TFSI)_2_‐[PY14]TFSI IL electrolyte could reduce the solubility of PTCDI and stabilize its structure. The as‐fabricated Mg‐DIBs presented excellent rate property and cycling performance with a capacity retention rate of 85% at 20 C (2000 mA g^−1^) and 95.7% at 5 C (500 mA g^−1^) after 500 cycles (Figure 10b,c).

#### 3.1.5 Zinc‐Ion Batteries

Among many emerging energy storage technologies in the post‐petroleum era, the battery system using Zn anode has received renewed attention in recent years.^[^ ^43^, ^139^ ^]^ The reason is that Zn has abundant sources and low cost.^[^ ^140^ ^]^ Zn has relatively low oxidation regenerating potential and is compatible with aqueous electrolytes. Great progress in various cathode materials of Zn batteries have been witnessed in recent years.^[^ ^141^, ^142^ ^]^ However, in the common alkaline electrolytes, Zn is not only vulnerable to corrosion, but also suffers surface passivation and dendritic growth after cycling.^[^ ^143^ ^]^ Moreover, the alkaline electrolytes also have volatilization issue and the toxicity of carbon dioxide, which greatly limit the charge–discharge performance and cycling life of secondary Zn batteries. Although the usage of acidic or neutral electrolytes has been proposed in recent years to improve the reversibility of Zn anode,^[^ ^140^, ^144^ ^]^ this system still faces several problems including continuous water consumption and low Coulombic efficiency caused by side reactions of Zn deposition and hydrogen evolution, as well as the dendrite formation on Zn anode resulting in poor cycling life and fast‐discharge performance. Therefore, it is very urgent to find efficient and low‐cost electrolytes to address these issues for practical applications. Clearly, nonaqueous ILs are very promising because several reports have already indicated that ILs can efficiently solve the dendrites of ZIBs and enhance the electrochemical properties.^[^ ^145^, ^146^, ^147^, ^148^, ^149^ ^]^

**Figure 11:**
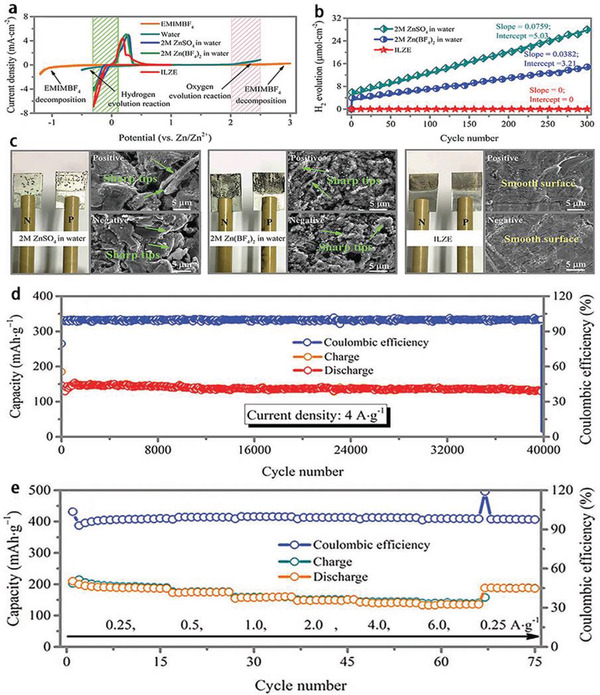
2 m ZnSO_4_, 2 m Zn(BF_4_)_2_ aqueous electrolytes and ILZE: a) electrochemical window of water and [EMIm]BF_4_ IL; CV curves of Zn plating/stripping using three‐electrode configuration (stainless steel foil as working and Zn foil as reference and counter electrodes), b) hydrogen production during Zn plating/stripping process at a current density of 0.5 mA cm^−2^, and c) the surface morphology of metallic Zn foils after 300 cycles at 0.5 mA cm^−2^. Electrochemical performance of a Zn/CoHCF battery: d) cyclic stability at 4 A g^−1^ and e) rate performance in ILZE. Reproduced with permission.^[^ ^78^ ^]^ Copyright 2020, Wiley‐VCH.

Very recently, the Zhi group^[^ ^78^ ^]^ reported the ZIBs with [EMIm]BF_4_‐Zn(BF_4_)_2_ electrolyte (named as ILZE) and CoHCF (cobalt hexacyanoferrate) cathode to solve the problems of hydrogen evolution reaction and Zn dendrites (**Figure** **11**a–c). The as‐fabricated ZIBs presented superb electrochemical stability, reflected in delivering over 40 000 cycles with 98% of capacity retention, excellent Coulombic efficiency around 100%, and ultrahigh rate performance owe to the high ionic conductivity of the IL‐based electrolytes (Figure 11d,e).

**Figure 12:**
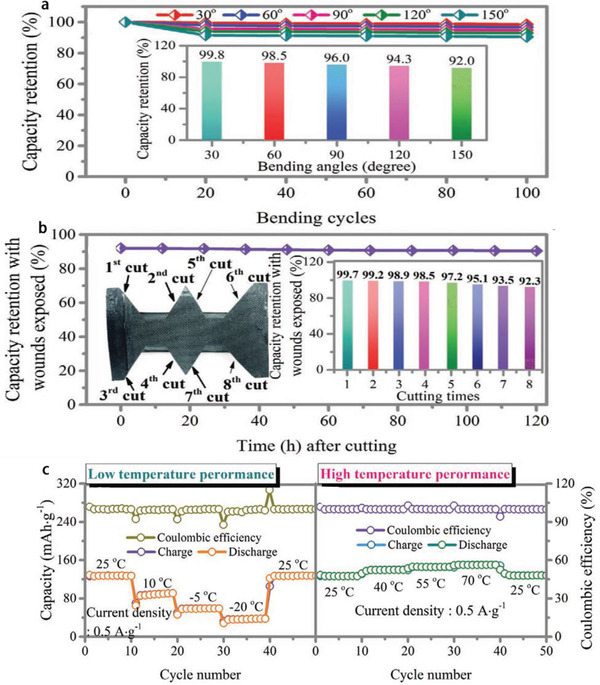
a) The all‐solid‐state Zn/CoHCF batteries under different angles at a fixed devices length of 5.5 cm, b) voltage of devices at different cutting status, and c) electrochemical performance at different temperatures from −20 to 70 °C at a current density of 0.5 A g^−1^. Reproduced with permission.^[^ ^78^ ^]^ Copyright 2020, Wiley‐VCH.

In addition, polymer electrolytes can also effectively inhibit the growth of Zn dendrites and the dissolution of active substances. Compared with aqueous electrolytes of ZIBs, polymer electrolytes can not only prevent liquid leakage, but also integrate the actions of diaphragm and electrolytes into one, which is helpful to simplify the ZIBs manufacturing process. However, their development is limited by low ionic conductivity, poor mechanical strength and high impedance at the interface between electrode and polymer electrolytes. Excitingly, some additives (such as ILs) are the promising candidates. Liquid‐free, flexible and mechanical properties of all solid‐state electrolytes based on polymers could make the batteries much safer and more widely used. The previous reports revolved that gel electrolytes are not at the level of all solid‐state batteries in the full sense.^[^ ^76^, ^150^, ^151^ ^]^ Furthermore, the Zhi group manufactured the PVDF‐HFP‐PEO‐ILZE all solid‐state electrolyte (named as PHP‐ILZE) (PEO: poly(ethylene oxide)) with ILZE as additive. The ILZE is contributed to offer Zn^2+^ and improve the ionic conductivity furtherly. The all solid‐state ZIBs obtained an excellent cycling performance (the life of batteries could exceed 30 000 cycles at 2 A g^−1^ at room temperature), flexibility and safety for 150° of bending deformation above 100 cycles and maintained normal working after being cut into many fragments for eight times (**Figure** **12**a,b), as well as wide applicable temperature from −20 to 70 °C (Figure 12c). This PHP‐ILZE electrolyte combines the advantages of solid electrolyte and the IL, and its ultra‐thin thickness (28.6 µm) ensures the ZIBs a high energy density. Moreover, the anions in the ILs could dynamically decrease the interfacial evolution process of Zn dendrites to some extent.^[^ ^146^ ^]^

ILs based solid‐state/gel electrolytes are usually adopt PVDF, PEO and HFP, etc. as electrolyte matrixes and ILs as additives, which are widely applied in ZIBs,^[^ ^152^ ^]^ LIBs,^[^ ^153^, ^154^ ^]^ SIBs,^[^ ^59^, ^60^ ^]^ and MIBs.^[^ ^155^, ^156^ ^]^

#### 3.1.6 Aluminum‐Ion Batteries

Among all metal elements in the earth's crust, Al is the most abundant one. Also, the conversion between Al atom and Al^3+^ cations involves the gain and loss of three electrons, which puts the theoretical capacity of AIBs (*C*_theo_ = 2980 mAh g^−1^) in the second position, only next to LIBs (*C*_theo_ = 3870 mAh g^−1^). Thus, the rechargeable AIBs are regarded as a promising stockpile device in future. However, the research on AIBs is very challenging and the progress is very slow because it is very difficult to find one candidate material that can maintain enough voltage after the repeated charge and discharge processes.

Since the carbon‐based electrode materials, ILs electrolytes, and their synergistic effect have obvious impacts on the performance improvement of the AIBs, many researches focusing on AlCl_3_‐type ILs electrolytes with graphite cathodes in AIBs are intensively reported.^[^ ^157^, ^158^, ^159^, ^160^ ^]^ In 2015, the Dai group^[^ ^161^ ^]^ made a breakthrough in rechargeable AIBs with a long lifespan of more than 7500 cycles and no loss of their capacity comparing to the instability of common AIBs after about 100 cycles. In their research, novel 3D graphitic foam cathode, Al anode and AlCl_3_/[EMIm]Cl IL electrolyte were employed to construct AIBs, where metal Al reacts with AlCl_4_^−^ to form Al_2_Cl_7_^−^ in the anode. The cathode mainly involving in the intercalation/deintercalation of AlCl_4_^−^ during the charge–discharge process. The 3D graphitic foam allows much faster diffusion and intercalation of AlCl_4_^−^ than pyrolytic graphitic, which could shorten the charge time to around one minute.

**Figure 13:**
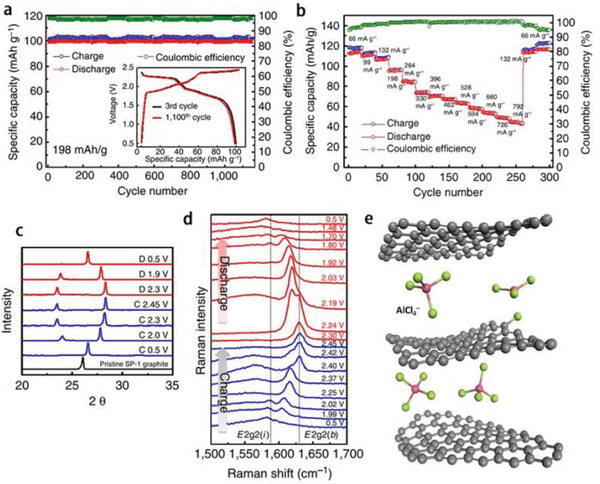
a) Long cycling performance of the AIBs at 198 mA g^−1^ between 0.5 and 2.45 V and b) capacity retention at various current densities, c) ex situ XRD patterns of NG in various charge and discharge states through the second cycle, d) in situ Raman spectra recorded for the NG cathode through a cycle showing chloroaluminate anion intercalation/deintercalation into graphite, and e) DFT model of chloroaluminate anions intercalating into the graphite layers. Reproduced with permission.^[^ ^162^ ^]^ Copyright 2017, Springer Nature.

Then in 2017, the Dai group^[^ ^162^ ^]^ reported their continuous research work to improve the performance of AIBs, where only one change is the usage of SP−1 natural graphite (NG) flakes film as the cathode. The authors believe that NG flakes film is outbalanced to synthetic graphite and could offer higher capacities and well‐defined voltage plateaus. The as‐fabricated AIBs did show two legible discharge plateaus in the ranges of 2.25–2.0, and 1.9–1.5 V, respectively. The discharge capacity was up to 110 mAh g^−1^ and maintained around 100 mAh g^−1^ at 198 mA g^−1^ over 1000 cycles with the Coulombic efficiency around 98% at 99 mA g^−1^. This performance is higher than that of pyrolytic graphite (≈60 mAh g^−1^). Even at a high current density of 6 C (660 mA g^−1^), the capacity and Coulombic efficiency remained 60 mAh g^−1^ and ≈99.5%, respectively (**Figure** **13**a,b). The in situ Raman spectra, X‐ray diffraction (XRD) patterns, and density functional theory (DFT) calculations have also been carried out to explore the intercalation reaction mechanism of chloroaluminate anions (AlCl_4_^−^ and Al_2_Cl_7_^−^) in the NG electrodes. The XRD patterns in Figure 13c were tested in the whole second cycle, where the (002) peak of pristine NG at 2*θ* = 26.05° gradually disappeared and then divided into two new peaks when charged to 2.0–2.45 V, representing the intercalation of chloroaluminate anions into NG. Afterward, the two peaks reverted to unimodal (26.6°) during the discharge process, representing the deintercalation process. These results clearly confirmed the highly invertible structure of NG during the intercalation/deintercalation processes. The in situ Raman spectra (Figure 13d) illustrated the intercalation/deintercalation processes. In addition, the four structures of tetrahedral AlCl_4_^−^ anions intercalated in the edge position of the graphite layers were furtherly investigated by DFT calculation (Figure 13e).

**Figure 14:**
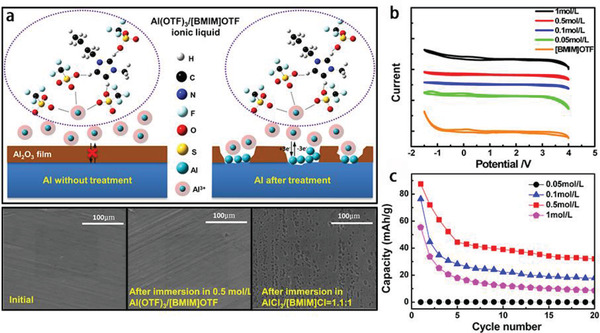
Schematic diagram of Al deposition/dissolution on surface of untreated/treated Al anode and SEM images of a) Al foils before and after immersion in Al(TfO)_3_/[BMIm]TfO and AlCl_3_/[BMIm]Cl for 24 h, b) electrochemical window, and c) cycling performance of AIBs with Al(TfO)_3_/[BMIm]TfO ionic liquids. Reproduced with permission.^[^ ^163^ ^]^ Copyright 2016, American Chemical Society.

Moreover, inorganic materials cathodes and ILs electrolytes can also be employed in AIBs. For example, V_2_O_5_ nanowirecathode can be applied in AIBs with AlCl_3_/[EMIm]Cl IL electrolyte (**Figure** **14**a).^[^ ^11^ ^]^ However, the AlCl_3_‐type ILs are corrosive to Al foil. To address this issue, Wang et al.^[^ ^163^ ^]^ presented a new strategy that the Al_2_O_3_ film was destroyed by AlCl_3_/[BMIm]Cl IL electrolyte to build a channel for Al^3+^ shuttling from Al anode, followed by employing the noncorrosive Al(TfO)_3_/[BMIm]TfO IL electrolyte to obtain stable interface of Al/electrolyte (Figure 14a). Note that the Al(TfO)_3_/[BMIm]TfO IL AIBs could achieve a wide charge–discharge voltage range with the highest potential of 3.25 V (the oxidation decomposition happened) (Figure 14b). The battery delivered an initial capacity of 87 mAh g^−1^ and stabilized at around 40 mAh g^−1^ at 10 mA g^−1^ (Figure 14c). These scientific payoffs reinforce the development advantages of AIBs in practice.

**Figure 15:**
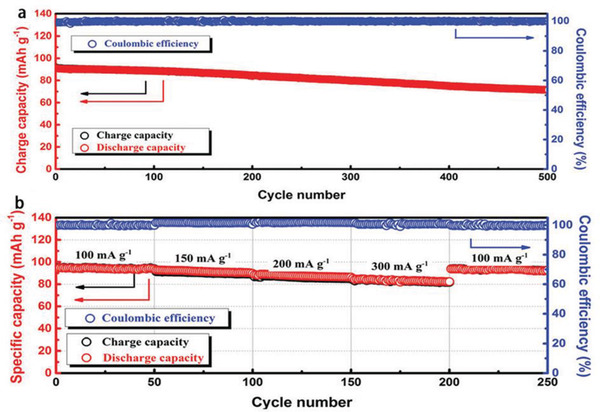
Characteristics of an AIB in AlCl_3_/urea = 1.5 electrolyte: a) long‐term cycling performance at 200 mA g^−1^ and b) rate property at varied current densities. Reproduced with permission.^[^ ^146^ ^]^ Copyright 2017, The Royal Society of Chemistry.

Besides the above‐mentioned IL systems, some cost‐effective molten electrolytes like AlCl_3_–urea systems have also been investigated in the AIBs, which displayed high efficiency and high stability.^[^ ^80^, ^164^, ^165^ ^]^ The battery mechanisms during the charge are Al deposition and the intercalation of AlCl_4_^−^ anion in graphite. When the molar ratio of AlCl_3_ to urea was less than 1.1, only AlCl_4_^−^ appeared. As the molar ratio of AlCl_3_ increased, Al_2_Cl_7_^−^ could form. If the molar ratio is controlled below 1.5, both AlCl_4_^−^ and Al_2_Cl_7_^−^ can be generated, however, if the ratio continues increasing, Al_3_Cl_10_^−^ will form in the electrolyte. Besides, the more AlCl_3_, the stronger the acidity of the electrolyte, which would cause the corrosion of Al foil and the fast capacity decay of AIBs.^[^ ^165^ ^]^ Later, the Dai group^[^ ^164^ ^]^ applied the cheap AlCl_3_–urea electrolyte with molar ratio of 1.3:1, and the as‐fabricated AIBs yielded a specific capacity around 73 mAh g^−1^ at 100 mA g^−1^ (≈1.4 C) after 180 cycles. At specific currents of 100 mA g^−1^ or 50 mA g^−1^, 87.8% and 90.0% of specific capacity were obtained with stable and high Coulombic efficiency >99%. The Jiao group^[^ ^165^ ^]^ prepared AlCl_3_/urea electrolyte with molar ratio of 1.5:1 for AIBs and also showed great potential at high temperature of 120 °C. The long‐term cycling test exhibited a capacity of 75 mAh g^−1^ at 200 mA g^−1^ (≈2.8 C) with a high Coulombic efficiency of 99% after 500 cycles (**Figure** **15**a). When the current density increased from 100 to 300 mA g^−1^, there was a slight drop of capacity and then nearly no fading as the cycling proceeded, indicating its excellent rate property (Figure 15b).

### 3.2 Applications of PILs Electrolytes

**Figure 16:**
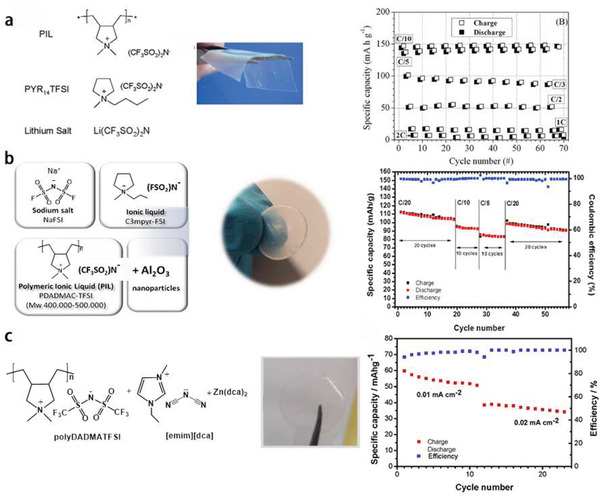
Compositions and cyclability of PIL‐based iongel electrolytes used in a) LIBs, b) SIBs, and c) ZIBs. a)Reproduced with permission.^[^ ^166^ ^]^ Copyright 2009, Elsevier B.V.b) Reproduced with permission.^[^ ^60^ ^]^ Copyright 2019, Elsevier B.V. c) c) Reproduced with permission.^[^ ^152^ ^]^ Copyright 2018, Elsevier Ltd.

PILs are one important classes of materials since they possess several interesting properties such as high thermal stability, good film forming ability, and good chemical compatibility with ILs.^[^ ^166^ ^]^ Compared with liquid electrolytes, PILs could suppress the dendrite formation due to their substantially low mobility. Though excellent electrochemical stabilities and ionic peculiarities make PILs good candidates as electrolyte hosts, the ionic fluidity decreases due to all monomers attached on the polymer chain. When ILs are added into PILs, they can act as the plasticizers to improve the ionic conductivity of PILs. In addition, introducing a certain amount of organic solvents (propylene carbonate (PC) or EC) into PILs can significantly reduce their viscosities.^[^ ^61^ ^]^ Moreover, the addition of nanoparticles can improve the ionic conductivity and increase the mechanical properties of the electrolyte.^[^ ^152^, ^167^ ^]^ Al_2_O_3_ is a commom particle.^[^ ^60^, ^154^ ^]^ **Figure** **16** displays the compositions and cyclability of three PIL‐based iongel electrolytes used in LIBs,^[^ ^166^ ^]^ SIBs,^[^ ^60^ ^]^ and ZIBs,^[^ ^152^ ^]^ respectively. These PILs are typically poly(diallyldimethylammonium) with FSI^−^ or TFSI^−^ as charge‐balanced species.

**Figure 17:**
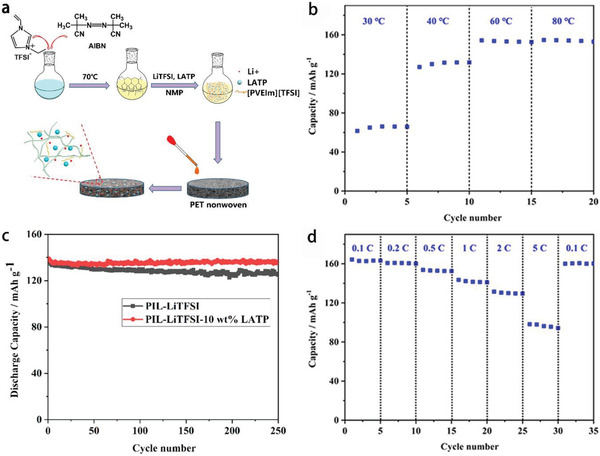
a) Schematic diagram of PET‐PIL‐LiTFSI‐LATP electrolyte preparation process, b) discharge capacity of LiFePO_4_/LIBs obtained at various temperatures at 0.5 C (85 mA g^−1^), c) cycling performance of LIBs with PIL‐based electrolyte at 60 °C at 1 C (170 mA g^−1^), d) discharge capacity of LIBs obtained at different rates at 60 °C. Reproduced with permission.^[^ ^167^ ^]^ Copyright 2019, American Chemical Society.

**Figure 18:**
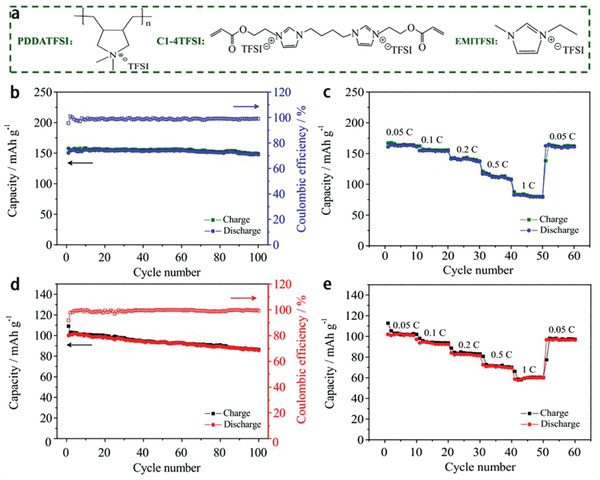
a) Various components of HPILSE, b,c) cycling and rate performance of LiFePO_4_/Li‐HPILSE LIBs at 0.1 C (17 mA g^−1^), d,e) cycling and rate performance of Na_0.9_[Cu_0.22_Fe_0.30_Mn_0.48_]O_2_/Na‐HPILSE SIBs at 0.1 C (10 mA g^−1^). Reproduced with permission.^[^ ^59^ ^]^ Copyright 2017, Elsevier Ltd.

Ma et al.^[^ ^167^ ^]^ prepared PIL with [VEIm]TFSI as monomer, LiTFSI as Li salt, nonwoven PET (polyethylene terephthalate) as rigid frame. This as‐prepared PIL together with ceramic electrolyte particles LATP (Li_1.3_Al_0.3_Ti_1.7_(PO_4_)_3_) as additive has been employed to fabricate LIBs. **Figure** **17**a displays the schematic diagram to prepare PET‐PIL‐LiTFSI‐LATP electrolyte. As the ionic conductivity of the PIL electrolyte increased with the increase of temperature, LIBs showed high‐capacity retention of 96.4% after 250 cycles at 1 C (170 mA g^−1^) and high‐rate property at 60 °C (Figure 17b–d). The added LATP can enhance the ionic conductivity, which makes LIBs more stable during cycling process. Zhou et al.^[^ ^59^ ^]^ synthesized PDDATFSI/poly(C1‐4TFSI)‐[EMIm]TFSI PIL electrolyte (HPILSE, **Figure** **18**a), which could be applied in both LIBs and SIBs. Poly(C1‐4TFSI) acted as crosslinked frameworks to prevent the leakage of [EMIm]TFSI electrolyte at high temperature, where the [EMIm]TFSI electrolyte was helpful to make the HPILSE possessed the high ionic conductivity and the PDDATFSI PIL could offer enough mechanical strength. When this complex was applied in the LiFePO_4_/LIBs, they could achieve a high discharge capacity of 147 mAh g^−1^ (97.7% of capacity retention) after 100 cycles at 0.1 C (17 mA g^−1^) with the Coulombic efficiency around 100% and great rate property of 81 mAh g^−1^ at 1 C (170 mA g^−1^) (Figure 18b,c). When PIL was used in the SIBs with Na_0.9_[Cu_0.22_Fe_0.30_Mn_0.48_]O_2_ electrode, the discharge capacity of the as‐fabricated SIBs could remain at 85.6 mAh g^−1^ (85.5% of capacity retention) after 100 cycles at 0.1 C (10 mA g^−1^) with the Coulombic efficiency around 100% and rate property of 60 mAh g^−1^ at 1 C (100 mA g^−1^) (Figure 18d,e).

## 4 Summary of Electrochemical Performances of Battery Systems

Systematically, the electrochemical properties of IL‐based electrolytes in various battery systems mentioned above are summarized and sorted in **Table** **3**. One can easily conclude that ILs with [RRIm]/[PYrr] as cations and FSI^−^, TFSI^−^, or BF_4_^−^ as anions are the most commonly used and most adaptable ones. They have been widely investigated in LIBs, SIBs, ZIBs, and MIBs. However, the reports of PIBs with ILs as electrolytes are rare, and so far, only [PYrr]FSI or [PYrr]TFSI were adopted in PIBs. As to ILs electrolytes in AIBs, AlCl_3_‐based ILs are most applicative and [EMIm]Cl ILs are most commonly used electrolytes, accounting for more than 60%. Some other IL electrolytes used in AIBs are [BMIm]X (about 10%), AlCl_3_–urea, and AlCl_3_–NaCl.

**Table 3:** Electrochemical properties of various IL‐based electrolytes used in different battery systems

Electrolyte	Initial capacity [mAh g^−1^]/current density	Rate performance [mAh g^−1^]/current density	Capacity retention [mAh g^−1^]/cycle number/current density	Systems & Ref
0.3 M LiTFSI‐[PY13]TFSI	390/0.2 C (89.2 mA g^−1^)	231/5 C (2.25 A g^−1^)	268 (68%)/1000/0.2 C	LIBs^[^ ^68^ ^]^
1 M LiPF_6_‐EC/DMC	423/0.1 C (44.6 mA g^−1^)	≈175/1 C (446 mA g^−1^)	216 (51%)/100/0.1 C	LIBs
0.3 M LiTFSI‐[PY13]TFSI	902/20 mA g^−1^		82%/100/50 mA g^−1^	LIBs^[^ ^101^ ^]^
1 M LiTFSI‐DOL/DME	≈275/50 mA g^−1^	382/500 mA g^−1^	≈130/20/50 mA g^−1^	LIBs
0.3 M LiTFSI‐[PY13]TFSI (65 vol%) with EC/DMC‐5%VC	≈155/0.1 C (17 mA g^−1^)	121/2 C (340 mA g^−1^)	≈157/30/0.1 C	LIBs^[^ ^99^ ^]^
LiTFSI‐[BMIM]BF_4_/PVDF‐HFP/PC/EC	130/2 C (340 mA g^−1^)		95.5%/300/2 C	LIBs^[^ ^75^ ^]^
PEO + LiTFSI‐[PY13]TFSI	≈158/0.1 C (17 mA g^−1^)		≈163/100/0.1 C	LIBs^[^ ^168^ ^]^
SiO_2_PPTFSI/PVDF‐HFP	119/1 C (130 mA g^−1^)	74/6 C (780 mA g^−1^)	92.1%/460/1 C	LIBs^[^ ^77^ ^]^
SBA‐15/LiTFSI‐[PYRA_12O1_]TFSI/PVDF‐HFP	≈150/0.05 C (8.5 mA g^−1^)	≈120/0.2 C (34 mA g^−1^)	124/174/0.2 C	LIBs^[^ ^74^ ^]^
0.3 M NaTFSI‐[PY13]TFSI	343/0.1 C (44.6 mA g^−1^)		≈342 (99.7%)/300/0.29 C (130 mA g^−1^)	SIBs^[^ ^66^ ^]^
0.3 M NaTFSI/DME			47/100/0.29 C (130 mA g^−1^)	SIBs^[^ ^66^ ^]^
0.3 M NaTFSI‐[PY13]TFSI	≈265/30 mA g^−1^	≈180/300 mA g^−1^	≈170 (94%)/100/300 mA g^−1^	SIBs^[^ ^66^ ^]^
0.3 M NaTFSI/DME	≈130/300 mA g^−1^		≈30/100/300 mA g^−1^	SIBs^[^ ^66^ ^]^
NaCl‐buffered AlCl_3_/[EMIm]Cl	≈115/50 mA g^−1^	70/500 mA g^−1^	≈96%/460/150 mA g^−1^	SIBs^[^ ^70^ ^]^
1 M NaClO_4_‐EC/DEC‐5%FEC			≈79/450/150 mA g^−1^	SIBs^[^ ^70^ ^]^
1 M NaFSI‐[PY13]FSI	117/30 mA g^−1^	70/1000 mA g^−1^	97%/100/100 mA g^−1^	SIBs^[^ ^169^ ^]^
0.4 M NaFSI‐[EMIm]FSI	105/0.1 C (11.8 mA g^−1^)	51/10 C (1.18 A g^−1^)	94 (90%)/200/2 C (236 mA g^−1^)	SIBs^[^ ^170^ ^]^
0.25 M NaPF_6_‐[BMIm]TFSI	107/0.1 C (11.8 mA g^−1^)		104 (97%)/40/0.1 C	SIBs^[^ ^125^ ^]^
0.3 M KTFSI‐[PY14]TFSI + 2 wt% ES	47/1 C (50 mA g^−1^)	≈45/5 C (250 mA g^−1^)	42/1500/5 C	PIBs^[^ ^130^ ^]^
1 mol kg^−1^ KFSI‐[PY13]FSI	111/0.1 C (15.5 mA g^−1^)	≈50/2 C (310 mA g^−1^)	87.4%/100/0.1 C	PIBs^[^ ^73^ ^]^
0.5 M KTFSI‐[PY13]TFSI	65/0.05 C (6.4 mA g^−1^)		≈94%/70/0.05 C	PIBs^[^ ^171^ ^]^
0.5 M KTFSI‐[PY13]TFSI	52/0.05 C (6.4 mA g^−1^)		≈96%/20/0.05 C	PIBs^[^ ^171^ ^]^
0.5 M KTFSI‐[PY13]TFSI	69/0.05 C (6.4 mA g^−1^)		≈94%/20/0.05 C	PIBs^[^ ^171^ ^]^
0.25 M POC‐0.2 [PY14]Cl	370/20 mA g^−1^	300/50 mA g^−1^	340/88/20 mA g^−1^	MIBs^[^ ^135^ ^]^
0.4 M Mg(TFSI)_2_‐[PY14]TFSI	57.7/2 C (200 mA g^−1^)	49.1/20 C (2 A g^−1^)	95.7%/500/5 C (500 mA g^−1^)	MIBs^[^ ^138^ ^]^
0.2 M Zn(TfO)_2_/[EMIm]TfO	≈34/0.2 mA g^−1^	≈22/0.5 mA g^−1^	≈18/100/0.2 mA g^−1^	ZIBs^[^ ^65^ ^]^
PVDF‐HFP‐PEO‐ILZE	187.3/0.25 A g^−1^	135.6/6 A g^−1^	≈98%/40 000/4 A g^−1^	ZIBs^[^ ^78^ ^]^
1 M Zn(TFSI)_2_ + 21 M LiTFSI‐polyacrylamide hydrogel	275/0.3 A g^−1^	105/6 A g^−1^	92%/5300/1 A g^−1^	ZIBs^[^ ^172^ ^]^
polyDADMATFSI‐Zn(dca)_2_ [EMIm][dca]/H_2_O/Al_2_O_3_	51/0.01 mA cm^−2^			ZIBs^[^ ^152^ ^]^
AlCl_3_‐[EMIm]Cl	≈70/1 C (66 mA g^−1^)	≈60/75 C (5 A g^−1^)	≈60(100%)/7500/60 C (4 A g^−1^)	AIBs^[^ ^161^ ^]^
AlCl_3_‐[EMIm]Cl	≈110/0.9 C (37.2 mA g^−1^)	≈60/6 C (660 mA g^−1^)	≈60/6000/10 C (1.1 A g^−1^)	AIBs^[^ ^162^ ^]^
AlCl_3_‐[EMIm]Cl	305/125 mA g^−1^		273 (90%)/20/125 mA g^−1^	AIBs^[^ ^11^ ^]^
AlCl_3_–urea‐[EMIm]Cl (13.5:9:0.8)	≈78/10 mA g^−1^	≈66/10 mA g^−1^	≈66/150/100 mA g^−1^	AIBs^[^ ^80^ ^]^
AlCl_3_–urea	≈73/100 mA g^−1^	≈64/200 mA g^−1^	≈72/200/100 mA g^−1^	AIBs^[^ ^164^ ^]^

## 5 Conclusions and Prospects

This review mainly summarizes the recent progress in the application of different kinds of ILs electrolytes in high performance metal‐ion batteries (Li, Na, K, Mg, Zn, and Al). ILs electrolytes have many advantages over ordinary organic electrolytes including their nonflammability, wide liquid temperature range, and high safety, which are essential in practical applications. Different ILs electrolytes, ILs additives, PILs, and ILs‐based solid/gel electrolytes have different contributions to the electrochemical performance of batteries. The introduction of ILs into metal‐ion batteries could ensure high safety and excellent cycling stability (over thousands even tens of thousands of cycles). The current main research on ILs should focus on the working mechanisms of various ILs and their compatibilities as well as the improvement of the inherent defects in various battery systems. For example, in SIBs, the large ionic radius of Na^+^ is an obstructive factor and the shuttle effect should be optimized, while in MIBs and AIBs, finding suitable electrode materials and searching exact electrolytes are the key issues. Besides, it is also important to reduce the occurrence of corrosion by optimizing the electrolytes. As to ZIBs systems, the main objectives are to inhibit the formation of Zn dendrites and develop suitable cathode materials. One should note that PIBs with ILs as electrolytes are still in their infancy and many efforts are required to further explore this system where electrode materials with high energy density are strongly required.

Although ILs are believed to have a promising future in overcoming most of these battery problems, their high cost has become an obstacle for their large‐scale applications. Moreover, the choices of electrolytes to be used in the batteries are limited. Thus, developing new ILs with low cost and high performance is highly desirable. One should note that the disadvantages of ILs can be compensated by the additional functional capabilities of batteries brought by ILs‐based electrolytes including ultra‐long durability, high safety and wide applicable temperature range. With the gradually mature technology, the ILs would have wide applications in practical rechargeable batteries.

## Conflict of Interest

The authors declare no conflict of interest.
